# Pneumococcal serotypes and their association with death risk in invasive pneumococcal disease: a systematic review and meta-analysis

**DOI:** 10.3389/fmed.2025.1566502

**Published:** 2025-05-14

**Authors:** Samuel Darkwah, Namwin S. Somda, Samiratu Mahazu, Eric S. Donkor

**Affiliations:** Department of Medical Microbiology, University of Ghana Medical School, Accra, Ghana

**Keywords:** invasive pneumococcal disease (IPD), *Streptococcus pneumoniae*, mortality, pneumococcal vaccine, serotype, case fatality, relative-risk

## Abstract

**Background:**

*Streptococcus pneumoniae* and its infections are a global public health concern. Invasive pneumococcal disease accounts for significant mortality in the aged and immunocompromised. Over 100 unique capsular serotypes have been identified, with 80–90% of invasive disease attributable to about 23 serotypes. Pneumococcal serotype influences invasiveness, virulence, carriage, and IPD outcome. Case fatality rates among different pneumococcal serotypes in IPD have been inconsistently reported, prompting the need for a comprehensive meta-analysis. We hypothesized that specific pneumococcal serotypes would be associated with higher case fatality rates and that non-vaccine serotypes may exhibit increased mortality risks over time.

**Methods:**

We conducted a systematic review and meta-analysis of serotype-specific risk of death due to invasive pneumococcal disease (IPD) in the last decade. We calculated the risk ratio (RR) and 95% confidence interval (CI) for each serotype compared with serotype 14 in each study. Pooled risk ratios were computed using random effects size model analysis. We also conducted heterogeneity testing and meta-regression sub-analysis.

**Results:**

In total, 45 eligible studies were included, and 16 were selected for meta-analysis. Study distribution showed a global disparity, with Europe as the major data source. Serotype 31 had the highest case fatality rate (31.4%), indicating a concerning mortality risk associated with this serotype, particularly in immunocompromised patients. Overall, IPD patients with serotypes 3, 6A, 11A, 15A, 19F, and 31 were more likely to die. In contrast, serotypes 1, 5, 7F, and 8 IPD isolates recorded a reduced risk ratio compared to serotype 14. Subgroup analysis showed that vaccine serotypes were associated with a greater risk of death than non-vaccine serotypes, but there were no significant differences in risk estimates between population groups.

**Conclusion:**

The study confirms the stable role of pneumococcal serotype in determining the clinical outcomes of invasive pneumococcal disease. Our findings underscore the importance of serotype-specific surveillance in IPD and call for the reconsideration of current pneumococcal vaccine formulations to address high-risk non-vaccine serotypes. Efforts to build research capacity, especially in low-resource regions such as Africa and South America, are highly recommended.

## 1 Introduction

*Streptococcus pneumoniae*, or pneumococcus, is an encapsulated bacterium popularly known to cause severe diseases among children and older people worldwide. According to the Global Burden of Disease study (a multinational collaboration, and comprehensive regional and global research program that investigates disease burden vis-a-vis risk factors, mortality and disability associated with major diseases), *Streptococcus pneumoniae* is the 3rd leading cause of death and is associated with the most deaths among children below the age of 5 years (1). The pneumococcal death burden is approximately a million per annum globally, primarily due to pneumonia, mostly in Africa and Asia (2, 3). *Streptococcus pneumoniae* is a major cause of otitis media, complicated pneumonia, meningitis, and septicemia/septic shock. The term “Invasive Pneumococcal Disease” (IPD) describes the more severe and invasive infections; this would include bacteremia, sepsis, meningitis, and osteomyelitis, or infections in which *S. pneumoniae* is usually isolated from a normally sterile site (e.g., blood, pleural fluid, joint fluid, peritoneal fluid, and cerebrospinal fluid) (4). The pathogenicity and virulence of *S. pneumoniae* lie within the antigenic polysaccharide capsule that protects the bacterium against host effectors and forms the basis of serotype classification (5). Over 100 unique serotypes are identified, with 80–90% of invasive disease attributable to about 23 serotypes (6). Clinical and epidemiological pneumococcal parameters such as invasiveness, virulence, carriage, and disease outcome are influenced by pneumococcal serotype (7, 8). Thus, different serotypes have been uniquely identified with specific IPD presentations and invasiveness. For example, serotypes 1, 2, 4, 5, 7F, 8, 9, 12F, 14, 16, 18C, 19A are more invasive, while 3, 6A, 6B, 11A, 15B/C, 19, and 23F are relatively less invasive. Concerning syndromes, serotypes 1 and 3 are more often isolated from patients with pneumonia, while serotypes 6, 10, and 23 are usually isolated from patients with meningitis. Globally, serotypes 14 and 19A are generally the most common isolates in IPD (7, 9).

Potent vaccines such as the pneumococcal conjugate vaccines (PCV 7, PCV10, PCV13) and the pneumococcal polysaccharide vaccine (PPSV23) protecting against 7, 10, 13, and 23 different serotypes are available for immunization against pneumococcal disease. PCV vaccination in children has reduced the incidence of IPD due to PCV serotypes. Herd immunity has also ensured reduced incidence among unvaccinated age groups. However, non-vaccine serotypes have since emerged in children and adults (10–12), precipitating the need for a revamped vaccine strategy. The potential change in the distribution of serotypes in the post-PCV era (an increase in the prevalence of non-vaccine serotypes) warrants the need for proper monitoring of serotypes and their associated mortality risks. This would help identify emerging non-vaccine serotypes and assess the impact of vaccine introduction. With the development of higher-valent vaccines such as PCV15 and PCV20 to broaden the coverage of pneumococcal vaccines, it is crucial to evaluate and understand the serotype-specific risk of death and the potential clinical benefits these vaccines provide. Such vital information would inform policy decisions regarding vaccine production, distribution and use.

Understanding the biological and clinical differences in specific pneumococcal serotypes can help mitigate the global burden of IPD in various ways such as improving vaccine development strategies, improving IPD outcome predictions, and guiding antimicrobial therapy for IPD. Specifically, our knowledge on the risk of death or mortality associated with specific pneumococcal serotypes can be helpful in predicting IPD outcomes to make more informed treatment decisions. Thus we conducted this systematic review and meta-analysis to assess the serotype-specific risk of death due to IPD, and to confirm whether or not the risk of death is a stable serotype-associated property. Additionally, our objective was to determine which non-vaccine serotypes with an elevated risk of death had emerged in the last decade (from 2010, when the last relevant study was published for pneumococcal pneumonia).

## 2 Methods

### 2.1 Study design

This was a systematic review and meta-analysis of the available studies on IPD and pneumococcal serotype case fatalities from 2010 to date.

### 2.2 Sources of data, inclusion criteria, and literature review

We conducted a literature search between October 17 and October 28, 2024, for relevant articles published from the year 2010 to the year 2024 in PubMed, Web of Science, Scopus, and Science Direct databases. Additionally, a search using the Google Scholar web search engine was performed. The combination of the search terms included “pneumococcus,” “pneumococcal,” “invasive pneumococcal disease,” “serotype,” “fatality,” “mortality,” and “severity.” Some precise search terms (including MESH terms) used were: (Pneumococcus OR Streptococcus-pneumoniae* OR “*Streptococcus pneumoniae*” [Mesh]) AND (Pneumococcal OR Pneumococcal-disease* OR “Pneumococcal”[Mesh]) AND (Invasive-pneumococcal-disease* OR “Pneumococcal Infections”[Mesh]) AND (Serotype* OR “Serogroup”[Mesh])

AND (fatality OR mortality OR “Mortality”[Mesh]) AND (severity) Filters: English, from 2010 to 2024.

We also reviewed the citation lists of published texts to identify relevant articles. Because we aimed to assess the association of serotypes with the risk of death in IPD specifically, studies published in the English language from 2010 to 2024 that reported serotype-specific data of IPD cases and deaths were considered. Studies were excluded if they were case studies or reviews, lacked contemporary diagnostic procedures in identification and serotyping, isolates were from the non-sterile site or non-invasive pneumococcal infections, non-serotype-specific (pooled/serogrouped) data, reported no fatalities or mortality data, did not contain extractable data, full text was not available, and study subjects were non-human. Some studies with good data were excluded because they had one or more of the aforementioned reasons for exclusion, including the possibility of overlaps.

**FIGURE 1:**
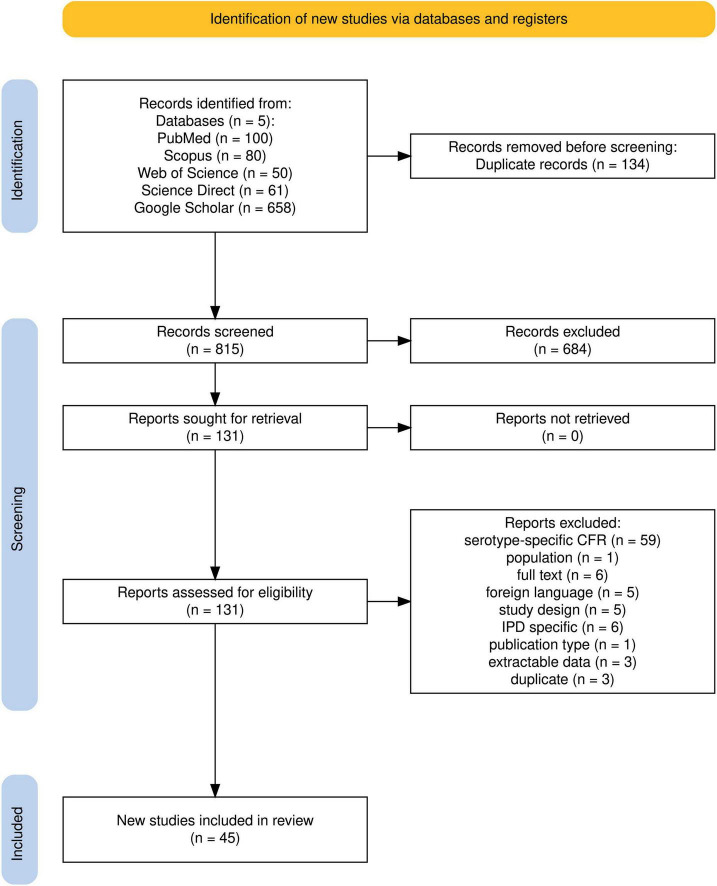
Flowchart of the systematic search and selection process. The flowchart was generated using the Shiny App online tool (SEI) (https://www.eshackathon.org/software/PRISMA2020.html) (43).

The literature search yielded 949 articles, which were exported, compiled, and imported into Rayyan AI and Endnote for management and deduplication. The titles and abstracts of deduplicated yields (815 articles) were reviewed and sorted using the web-based version of Rayyan AI. The full text of 131 articles selected from the abstract screening was thoroughly read and subjected to further screening, and relevant articles were selected for the study. A total of 45 articles were included in the study (Figure 1; Table 1).

**TABLE 1:** Overview of study characteristics included in the systematic review and meta-analysis.

Authors	Title	Country	Study period	Population	Sample size
Ahl et al. (44)a	High incidence of septic shock caused by Streptococcus pneumoniae serotype 3-a retrospective epidemiological study	Sweden	2006–2008	Adults	513
Alexandrova et al. (45)	Mortality of Invasive Pneumococcal Disease following Introduction of the 13-Valent Pneumococcal Conjugate Vaccine in Greenland	Greenland	1995-2020	All ages	295
Amin-Chowdhury et al. (46)	Characteristics of Invasive Pneumococcal Disease Caused by Emerging Serotypes After the Introduction of the 13-Valent Pneumococcal Conjugate Vaccine in England: A Prospective Observational Cohort Study, 2014–2018	England	2014-2018	All ages	21,592
Amin-Chowdhury et al. (47)	Invasive pneumococcal disease due to 22F and 33F in England: A tail of two serotypes	England	2014-2019	All ages	27,208
Aydin et al. (48)	Microbiological and clinical characteristics of Streptococcus pneumoniae serotype 3 infection and risk factors for severe outcome: A multicenter observational study.	Taiwan	2012-2021	All ages	146 (total) (20 invasive)
Ba et al. (49)	Pediatric invasive pneumococcal disease in Senegal	Senegal (Dakar)	2008-2013	Children	218
Beatty et al. (50)^a^	Prognostic factors associated with mortality and major in-hospital complications in patients with bacteremic pneumococcal pneumonia Population-based study	Canada	2000-2010	Adults	1636
Calvo-Silveria et al. (51)	Evolution of invasive pneumococcal disease by serotype 3 in adults: a Spanish three-decade retrospective study	Spain, Barcelona	1994-2020	Adults	270
Castro et al. (52)^a^	Invasive Pneumococcal Disease Characterization in Adults and Subgroups aged < 60 years and ≥ 60 years in Bogota, Colombia	Colombia	2011-2017	Adults	169
Ciruela et al. (15)^a^	Indirect effects of pediatric conjugate vaccines on invasive pneumococcal disease in older adults	Spain, Catalonia	2014-2016	Adults	1285
Cohen et al. (53)^a^	Streptococcus pneumoniae serotypes and mortality in adults and adolescents in South Africa: Analysis of national surveillance data, 2003-2008	South Africa	2003-2008	Adults	3952
Cremers et al. (54)^a^	Effects of 7-valent pneumococcal conjugate 1 vaccine on the severity of adult 2 bacteremic pneumococcal pneumonia	Netherlands	2001-2011	Adults	343
De Miguel et al. (27)^a^	Age-dependent serotype-associated case-fatality rate in invasive pneumococcal disease in the autonomous community of Madrid between 2007 and 2020	Madrid, Spain	2007-2020	All ages	6013
Gaensbauer et al. (55)	Pediatric Invasive Pneumococcal Disease in Guatemala City: Importance of Serotype 2	Guatemala	1996-2007	Children	452
Grau et al. (56)^a^	Declining mortality from adult pneumococcal infections linked to children’s vaccination	Barcelona,Spain	1994-2013	Adults	1811
Hanada et al. (57)	Multiple comorbidities increase the risk of death from invasive pneumococcal disease under the age of 65 years	Japan	2010-2017	All ages	581
Houseman et al. (58)^a^	Decreasing case fatality rate following invasive pneumococcal disease, North East England, 2006–2016	North East England	2006-2016	All ages	2510
Hsu et al. (59)	Changing serotypes causing childhood invasive pneumococcal disease: Massachusetts, 2001-2007	Massachusetts, USA	2001-2007	Children	586
Hu et al. (60)	Health and economic burden of invasive pneumococcal disease associated with 15-valent pneumococcal conjugate vaccine serotypes in children across eight European countries	Multinational (Europe-Denmark, France, Germany, Italy, Norway, Spain, Switzerland, UK)	2018 cohort	Children	3.2mil
Hu et al. (61)	Health and economic burden associated with 15-valent pneumococcal conjugate vaccine serotypes in children in the United States	USA	2018 cohort	Children	estimated 18,983
Inverarity et al. (62)	Death or survival from invasive pneumococcal disease in Scotland: associations with serogroups and multilocus sequence types	Scotland	1992-2007	Not mentioned	5959
Lai Chen-Yin et al. (63)	Comparison of invasive pneumococcal disease caused by serotype 19A and non-19A pneumococci in children: More empyema in serotype 19A invasive pneumococcal disease	Taiwan	2007-2011	Children	56; cases(27) control (29)
Lujan et al. (64)^a^	Influence of pneumococcal serotype group on outcome in adults with bacteraemic pneumonia.	Spain	1999-2009	Adults	299
Makwana et al. (65)	Pneumococcal-related hemolytic uremic syndrome in the United Kingdom: national surveillance, 2006–2016	England	2006-2016	Children	54
Marrie Thomas et al. (66)	Invasive pneumococcal disease in Northern Alberta, not a Red Queen but a dark horse.	Canada	2000-2014	All ages	509 (children), 2417 (adults)
Martinez-Vega et al. (67)	Risk factor profiles and clinical outcomes for children and adults with pneumococcal infections in Singapore: A need to expand vaccination policy?	England	1997-2013	All ages	889
Müller et al. (16)^a^	Streptococcus pneumoniae Serotypes Associated with Death, South Africa, 2012-2018	Canada	2012–2018	All ages	6,865
Navarro-Torné et al. (68)	Risk factors for death from invasive pneumococcal disease, Europe, 2010	Multinational [Europe:-Austria, Belgium, Bulgaria, Cyprus, Czech Republic, Denmark, Estonia, Finland, France, Greece, Hungary, Iceland, Ireland, Italy, Latvia, Lithuania, Malta, Netherlands, Norway, Poland, Romania, Slovakia, Slovenia, Spain, Sweden, and United Kingdom)	2010	All ages	2,921
Oligbu et al. (69)	Effect of pneumococcal conjugate vaccines on pneumococcal meningitis, England and Wales, July 1, 2000-June 30, 2016	England and Whales	2006-2014	Children	3146
Oligbu et al. (70)	Childhood Deaths Attributable to Invasive Pneumococcal Disease in England and Wales, 2006-2014	England and Whales	2000-2016	All ages	84,473
Rioseco et al. (71)	[Bacteremic pneumococcal pneumonia in adults admitted to a general hospital. Experience in 60 cases].	Chile	2010-2024	Adults	70
Rock et al. (72)	Epidemiology of invasive pneumococcal disease and vaccine provision in a tertiary referral center	Ireland	2006-2010	All ages	122
Shimbashi et al. (73)	Epidemiological and clinical features of invasive pneumococcal disease caused by serotype 12F in adults, Japan	Japan	2013-2018	Adults	1277
Skoczyńska et al. (74)	Recent trends in epidemiology of invasive pneumococcal disease in Poland	Poland	2011-2013	All ages	1190
Stanek et al. (75)^a^	A 32-Year Study of the Effect of Pneumococcal Vaccines on Invasive Streptococcus pneumoniae Disease	USA	1983-2014	All ages	1196
Thomas et al. (76)	An explosive outbreak of Streptococcus pneumoniae serotype-8 infection in a highly vaccinated residential care home, England, summer 2012	England	August, 2012	Adults	23
van Hoek et al. (77)	Effect of serotype on focus and mortality of invasive pneumococcal disease: Coverage of different vaccines and insight into non-vaccine serotypes	England and Whales	April 2002 to March 2011	All ages	23,688
Verhaegen et al. (78)	Epidemiology and outcome of invasive pneumococcal disease among adults in Belgium, 2009–2011	Belgium	2009-2011	Adults	1332
Vestjens et al. (79)	Twelve years of pneumococcal conjugate vaccination in the Netherlands: Impact on incidence and clinical outcomes of invasive pneumococcal disease	Netherlands	2004-2018	All ages	8865
Von Mollendorf et al. (80)	Epidemiology of serotype 1 invasive pneumococcal disease, South Africa, 2003-2013	South Africa	2003-2013	All ages	46,483
Wagenvoort et al. (81)^a^	Invasive pneumococcal disease: Clinical outcomes and patient characteristics 2-6 years after introduction of 7-valent pneumococcal conjugate vaccine compared to the pre-vaccine period, the Netherlands	Netherlands	2004-2012	All ages	4,853
Wu Shuiyan et al. (38)^a^	Early clinical predictors for the prognosis of invasive pneumococcal disease	China	2011-2017	Children	97
Xu et al. (39)^a^	Clinical characteristics and serotype distribution of invasive pneumococcal disease in pediatric patients from Beijing, China	China	2014-2019	Children	68
Yeon Jung et al. (82)^a^	Impact of national pneumococcal vaccination program on invasive pneumococcal diseases in South Korea	South Korea	2014-2019	All ages	893
Zurawska et al. (83)	Outcomes of Critically Ill Patients Who Have Serotype 5 Invasive Pneumococcal Disease	Canada	2004-2007	All ages	149

Characteristics of 45 studies included in the study. (a) Studies that were eligible for meta-analysis (included extractable serotype-specific cases and deaths; included serotype 14 data; reported serotypes well represented with at least 10 isolates per serotype).

### 2.3. Data extraction and analysis

Data from screened publications were extracted and managed in macOS Numbers spreadsheets under multiple headings, such as study, study design, year of publication, period/duration of study, location, region/country, sample size, population type, IPD syndrome, sample type, isolation procedure, serotyping technique, isolation site, serotypes, cases per serotype, deaths per serotype, and overall case fatality. We performed descriptive statistics on the extracted data, and meta-analysis on some selected studies using RStudio Software, Jamovi, and macOS Numbers.

### 2.4 Selection for meta-analysis and statistical analysis

Only serotypes found in at least three studies, with a minimum of 10 isolates per study, were considered for meta-analysis. Pooled estimates using the random effects model, heterogeneity testing, and meta-regression sub-analysis were conducted on 16 studies that met the criteria and recorded case fatalities for serotype 14 in the dataset. We calculated the risk ratio (RR) and 95% CI for each serotype compared with serotype 14 in each study. We chose serotype 14 as the reference serotype because it is the most commonly invasive serotype and, thus, the most frequent cause of IPD (13). Additionally, serotype 14 contained non-zero death entries across the studies we identified. Some serotypes had no fatalities in some studies. Hence, a value of 0.5 was added to each component of the RR for continuity before calculation.

Pooled RR for each serotype was computed by a random effects size estimate model (REML) using the “meta” and “metaphor” packages in RStudio (by Posit Software, PBC, Version 2024.12.0+467). The random effects model (REML) accounts for variability between studies and ensures more generalized findings. Heterogeneity among the studies was evaluated using the I^2^ approach to estimate the percentage of variability among studies that can be attributed to true heterogeneity rather than random variation. An I^2^ percentage above 25% is considered low-level heterogeneity, while I^2^ of 50% and 75% are moderate and high heterogeneity, respectively (14). A high I^2^ may indicate that there could be significant differences in the results from the combined studies, hence caution must be taken in interpreting the pooled findings (thus, taking into consideration the reasons for the variations in the individual studies). The level of significance was set at *p* < 0.05. All figures, tables, and plots were generated as part of the analysis in RStudio and macOS Numbers using the respective packages and libraries.

## 3 Results

### 3.1 Overview and distribution

**FIGURE 2:**
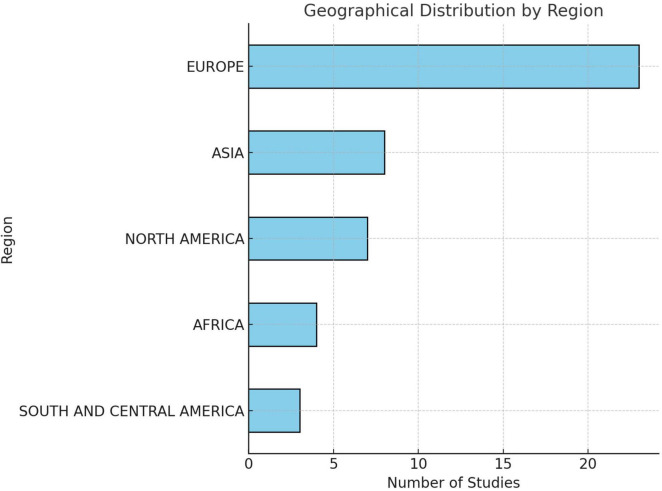
Geographical distribution of studies reporting serotype-specific case fatalities in IPD. Figure shows a global distribution skewed to Europe and Asian countries, and an uneven representation of focus data.

A total of 45 studies from 5 major geographical regions were analyzed. Europe accounted for the majority, with 23/45 studies (51.1%), followed by Asia with 8/45 studies (17.8%) and North America with 7/45 studies (15.6%). Africa and South and Central America were underrepresented, contributing only 4/45 studies (8.9%) and 3/45 studies (6.7%), respectively. England was the most frequently studied country, contributing 4/45 studies (8.9%), followed by the Netherlands, South Africa, and Canada, each contributing 3/45 (6.7%) studies. The remaining studies were distributed among other countries, including Taiwan, China, and the United States, highlighting an uneven, broad geographical representation (Figure 2).

The studies were published between 2010 and 2024, with the most frequent publication year being 2019 (7 studies, 15.6%). The mean publication year was 2017.4 (SD: 3.7), and the median was 2018. Study periods (in years) ranged from 1983 to 2018. The mean starting year was 2005.6 (SD: 7.5), and the median starting year was 2006.

The population groups varied widely across the studies. A mixed population, including all age groups, was most frequent. There were 4 (8.9%) and 11 (24.4%) studies that investigated adults and children, respectively (Table 1). The “children” studies had various subcategories, such as newborns, children under 5 years, and children under 18. One study reported findings exclusively from the aged (>64 years) (15). The IPD syndromes were mainly pneumonia, meningitis, bacteremia/sepsis, and a combination of bacteremia with pneumonia or meningitis across the studies.

### 3.2 Meta-analysis

Sixteen (16) studies were included in the meta-analysis. Two studies, representing 12.5%, were pediatric studies, while 8/16 studies (50%) were that of adults above 17 years. Six studies (37.5%) reported on all age groups (Table 1). Serotype 19F had the highest representation of 14 studies (aside from the reference serotype 14 reported in all 16 studies). This was followed by serotypes 3 and 19A, each represented in 13/16 studies. Serotype 17F had the least representation (3 studies) (Supplementary Table S1).

**FIGURE 3:**
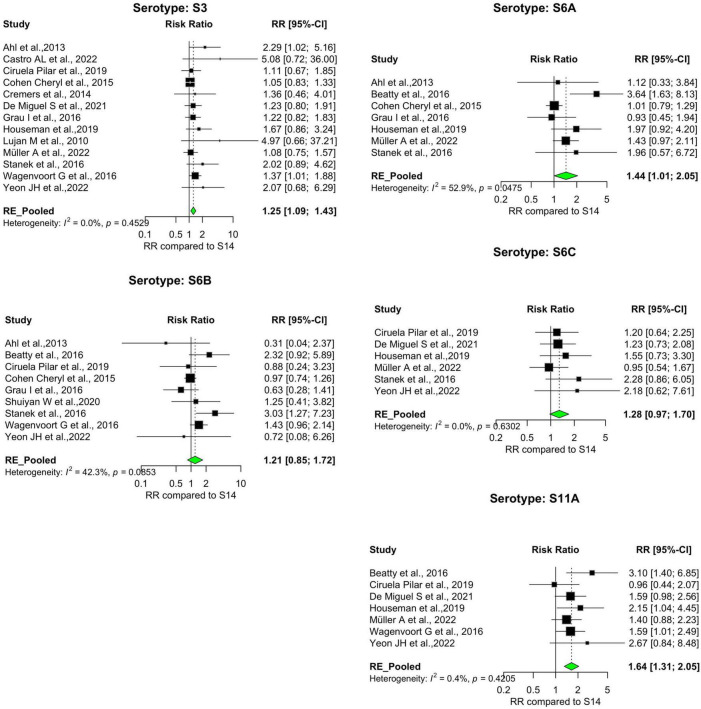
IPD serotypes with elevated risk of death. Study-specific and pooled risk ratios (RR) for death due to IPD compared with serotype 14. Black squares represent study-specific RR at 95% CI. Green diamonds represent the pooled RR (95% CI). I^2^ denotes the amount of variation in the RR due to heterogeneity across 16 studies.

**FIGURE 4:**
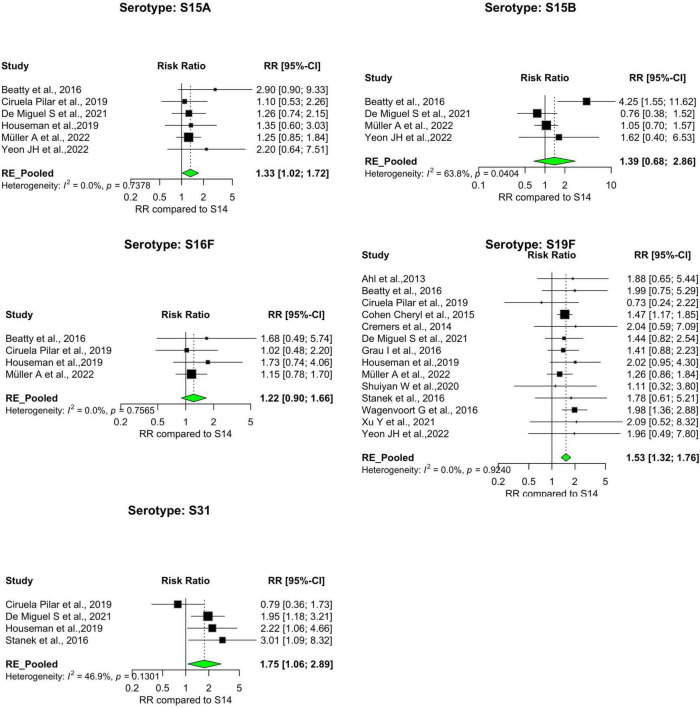
IPD serotypes with elevated risk of death_(continued). Study-specific and pooled risk ratios (RR) for death due to IPD compared with serotype 14. Black squares represent study-specific RR at 95% CI. Green diamonds represent the pooled RR (95% CI). I^2^ denotes the amount of variation in the RR due to heterogeneity across 16 studies.

**FIGURE 5:**
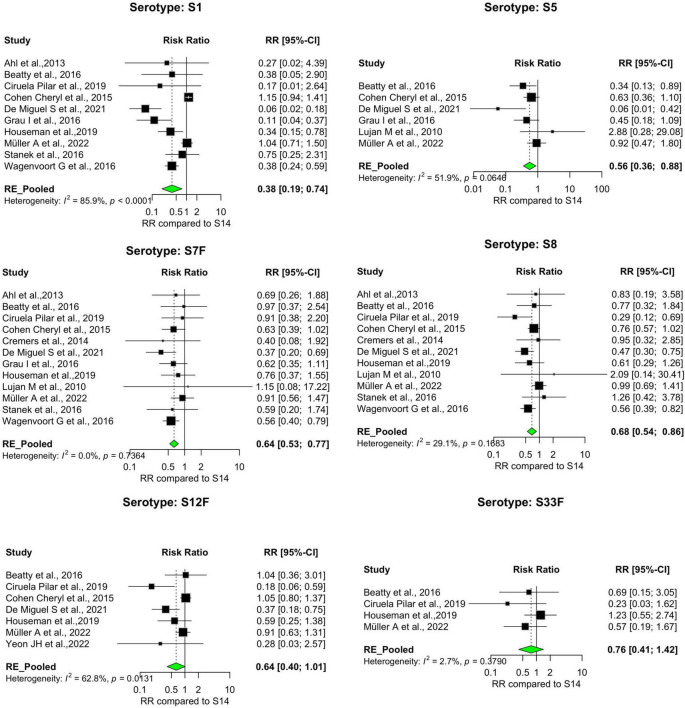
IPD serotypes with relatively lower risk of death. Study-specific and pooled risk ratios (RR) for death due to IPD compared with serotype 14. Black squares represent study-specific RR at 95% CI. Green diamonds represent the pooled RR (95% CI). I^2^ denotes the amount of variation in the RR due to heterogeneity across 16 studies.

**FIGURE 6:**
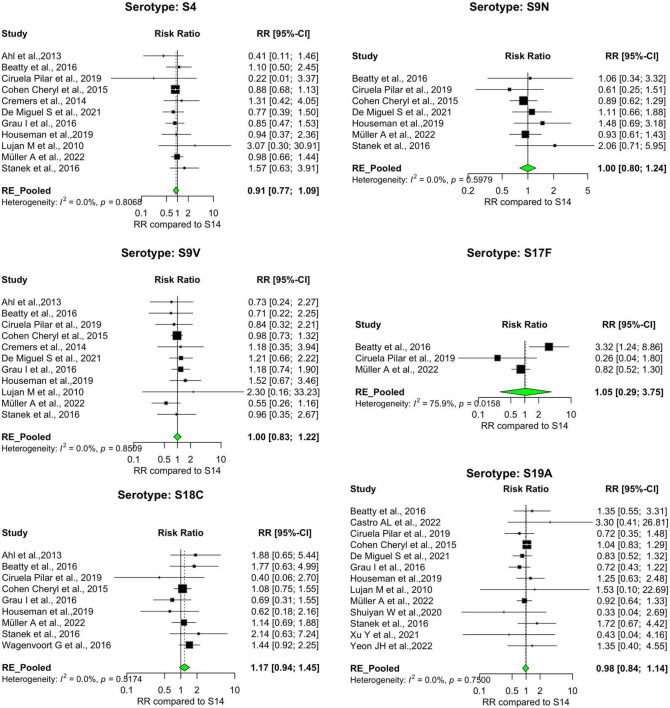
IPD serotypes with risk of death similar to reference serotype. Study-specific and pooled risk ratios (RR) for death due to IPD compared with serotype 14. Black squares represent study-specific RR at 95% CI. Green diamonds represent the pooled RR (95% CI). I^2^ denotes the amount of variation in the RR due to heterogeneity across 16 studies.

**FIGURE 7:**
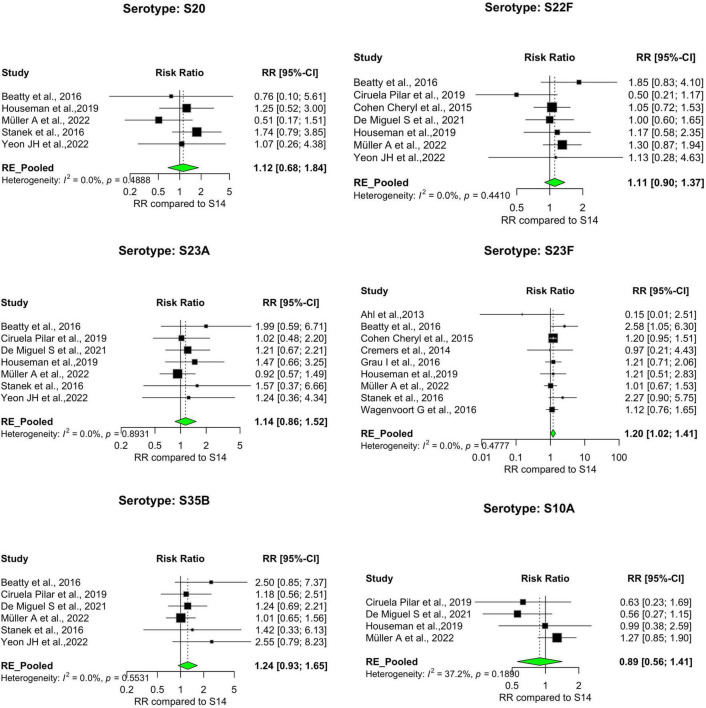
IPD serotypes with risk of death similar to reference serotype_(continued). Study-specific and pooled risk ratios (RR) for death due to IPD compared with serotype 14. Black squares represent study-specific RR at 95% CI. Green diamonds represent the pooled RR (95% CI). I^2^ denotes the amount of variation in the RR due to heterogeneity across 16 studies.

Our analysis revealed significant differences in the pooled estimates among the serotypes reviewed. Overall, IPD patients with serotypes 3, 6A, 11A, 15A, 19F, and 31 were more likely to die than patients with serotype 14. Other serotypes, including 6B, 6C, 15B, 16F, 18C, 20, 22F, 23A, and 35B, had pooled risk ratios greater than 1, suggesting a moderately higher risk of death. However, the estimates were not statistically significant (Figures 3, 4). Conversely, serotypes 1, 5, 7F, and 8 IPD cases were significantly less likely to die compared to those with serotype 14. Serotypes 20, and 12F, were drastically below the relative likelihood of death, although this was not statistically significant (Figure 5; Table 2). Studies focusing on pediatric IPD reported fewer serotypes (19A, 19F, 6B) than other study groups. Additionally, serotype 19A in all children-focused studies was low risk, while serotypes 19F and 6B were moderately high risk, although there was no statistical significance. Serotypes 9N and 9V had a pooled risk estimate of 1, which coincided with the reference serotype 14 (Figures 6, 7). There were low levels of heterogeneity among studies based on the I^2^ values for most serotypes. Serotypes 1, 5, 6A, 12F, 15B, 17F, and 31 exhibited moderate to high levels of heterogeneity.

**TABLE 2:** Serotype-specific risk of death due to IPD in the last decade.

	Serotypes		
	**Pooled RR < 1**	**Pooled RR ∼ 1**	**Pooled RR > 1**
Significant	1, 5, 7F, 8	23F	3, 6A, 11A, 19F, 31
Not significant	12F, 33F	4, 9N, 9V, 17F, 18C, 19A, 20, 22F, 23A, 35B	6B, 6C, 15A, 15B, 16F

Risk ratios of each serotype were computed with serotype 14 as a reference. Serotype-specific pooled RR estimates were done using the Random effects estimate model in 16 selected studies. RR < 1 = low risk of death, RR∼1, risk of death similar to reference, RR > 1 = high risk of death. Significance was defined as 95% CI that did not include 1

### 3.3 Case fatalities

Case fatality rates (CFRs) among pneumococcal serotypes provide crucial insights into disease severity and mortality risks. For each serotype, the total number of cases and deaths were used to calculate the case fatality rate (CFR). The CFR of the serotypes across the different studies was variable. Serotype 31 exhibited the highest average CFR of 31.4%, with 58 deaths among 195 cases. Serotype 11A showed a CFR of 30.1%, with 136 deaths reported out of 478 cases. Serotype 6A had a CFR of 29.7%, with 182 deaths from 530 cases. Serotype 19F, a commonly studied serotype, demonstrated a CFR of 28.6%, with 293 deaths from 836 cases. Other serotypes with notable CFRs included Serotype 3, which is often associated with invasive disease and shows moderate CFRs. The average CFRs across the studies for serotypes 33F, 5, 7F, and 1 were the lowest (12.8, 12.7, 12.4, and 9.9%, respectively) (Table 3).

**TABLE 3:** Average case fatality rates (CFR) of serotypes.

Serotype	Deaths (sum)	Cases (sum)	Av. CFR (%)
S31	58	195	31.35
S11A	136	478	30.15
S6A	182	530	29.74
S19F	293	836	28.58
S3	600	2327	27.55
S16F	82	253	26.46
S15A	115	408	26.29
S6C	84	355	25.94
S15B	67	275	25.41
S35B	76	299	24.35
S23F	217	765	22.81
S18C	83	336	22.07
S9N	121	540	21.68
S22F	161	745	21.66
S23A	71	329	21.23
S17F	35	146	20.93
S6B	126	496	20.70
S10A	68	301	20.02
S9V	125	601	17.88
S19A	418	1859	17.68
S4	221	1049	15.94
S20	58	293	15.76
S12F	196	998	13.65
S8	374	2716	13.34
S33F	18	122	12.80
S5	35	509	12.69
S7F	179	1660	12.40
S1	385	2154	9.89

The average case fatality rate (CFR) for each serotype was calculated as the ratio of the average total deaths to the average total cases [(Av.Deaths/Av.Cases) * 100]. The serotype with the highest average CFR was serotype 31 and the lowest was serotype 1.

### 3.4 Subgroup analysis by VT and NVT

**FIGURE 8:**
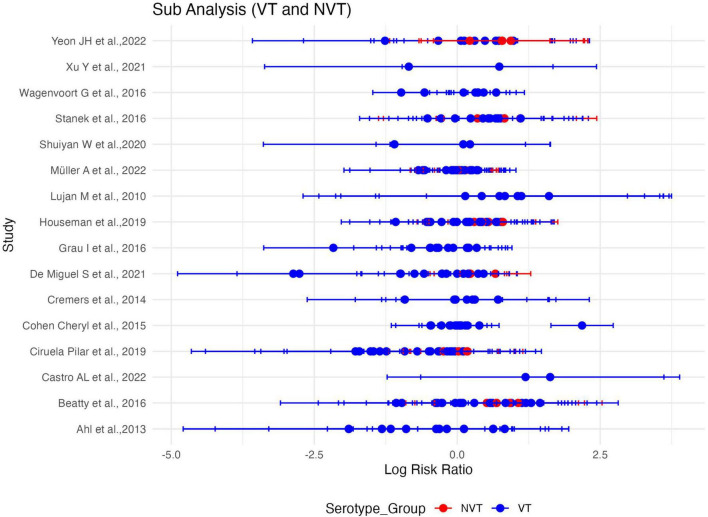
Sub-group meta-analysis assessing the difference in serotype-specific relative risk estimates of vaccine serotypes (VT) and non-vaccine serotypes (NVT). Plot of serotype-specific risk ratios across all sixteen (16) studies included in the meta-analysis, stratified by vaccine serotype (VT) and non-vaccine serotype (NVT) groups. Blue dot plots, demarcate vaccine serotypes and red plots demarcate non-vaccine serotypes.

**FIGURE 9:**
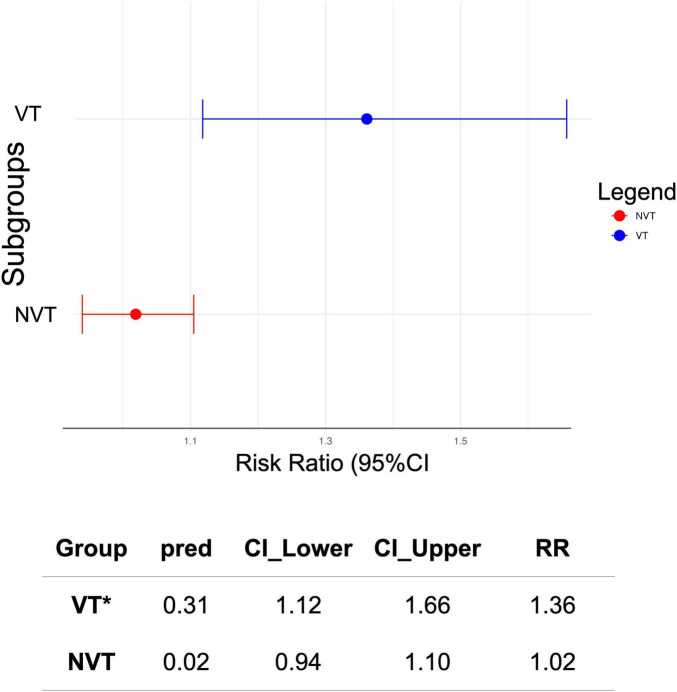
Predicted effect size (subgroup meta-analysis) and difference observed in risk ratios for vaccine (VT) and non-vaccine (NVT) serotypes. Pooled estimates of serotype-specific risk ratios across all sixteen (16) studies included in the meta-analysis, stratified by vaccine serotype (VT) and non-vaccine serotype (NVT) groups. (*) Higher risk of death associated with VT. Confidence intervals for VT and NVT were largely non-overlapping, hence, a meaningful difference between the two group.

We performed a meta-regression analysis to assess the relative risk (RR) of death associated with the different *S. pneumoniae* serotypes. Serotypes were categorized into vaccine serotypes (VT) and non-vaccine serotypes (NVT). The test for subgroup differences revealed a statistically significant difference between the two groups (*p* = 0.0076). The pooled RR for vaccine serotypes (VT) was 1.36 [95%CI: 1.12–1.66], indicating a significantly higher risk of death associated with the PCV and PPSV serotypes. In contrast, the pooled relative risk for non-vaccine serotypes (NVT) was 1.019 [95% CI: 0.939–1.105], suggesting no statistically significant risk of death in this group. The confidence intervals for VT and NVT were largely non-overlapping, further supporting a meaningful difference between the two groups. In summary, vaccine serotypes were associated with a higher risk of death than non-vaccine serotypes (Figures 8, 9).

### 3.5 Subgroup analysis by population groups

Separate random effects models were fitted for each population group. For the “Adults” subgroup, the estimated log RR was 0.0389 [95%CI: -0.0867 to 0.1645, *p* = 0.5441], with τ^2^ = 0.1510 and I^2^ = 49.61%, indicating substantial heterogeneity. For the “All Ages” subgroup, the estimated log RR was 0.0721 [95%CI: -0.0229 to 0.1671, *p* = 0.1371], with τ^2^ = 0.0844 and I^2^ = 37.34%, indicating moderate heterogeneity. In contrast, the Children subgroup showed no evidence of heterogeneity (τ^2^ = 0, I^2^ = 0%), and the RR was similarly not significant [logRR = 0.0539, 95%CI: -0.7327 to 0.8405, *p* = 0.8931]. The test for subgroup differences (Q_*M*_ = 0.1799, df = 2, *p* = 0.9140) revealed no statistically significant differences between the population groups. Across all scenarios of group exclusion in our sensitivity analysis, the pooled effect estimates remained statistically non-significant, demonstrating the robustness of the results.

## 4 Discussion

This systematic review and meta-analysis aimed to assess the serotype-specific risk of death due to IPD from 2010. We found that the risk of death varied by serotype in IPD. Several *S. pneumoniae* serotypes for both vaccine and non-vaccine types are associated with an increased risk of fatal outcomes however, vaccine serotypes significantly associate the risk of death more compared to non-vaccine serotypes. The elevated risk of death associated with serotypes 3, 6A, 11A, 15A, 19F, and 31 from our study is comparable to previous findings (7, 16, 17), however, we did not identify serotypes 9N and 6B as part of the elevated risk serotypes like previously reported by Weinberger et al. (7). What is interesting about this finding is the non-vaccine serotypes 31 and 15A, which are considered rarely and moderately invasive (13), respectively. These tend to have an increased risk of death compared to the commonly invasive serotype 14. This observation could support the inverse relationship between serotype invasiveness and disease severity or outcome. It is worth mentioning that invasiveness is a serotype-stable property (18).

Consistent with global studies and previous findings, serotype 1 (with less than 10% av. CFR) is often associated with lower CFRs and favorable disease outcomes in IPD outbreaks compared to serotypes like serotype 3 or 19F (7, 18). In line with this, we found that serotype 1 also had a significantly lower risk of death (Pooled RR = 0.38 [95%CI = 0.19–0.74]). Rarely reported serotypes such as serotype 33F and serotype 20 are known to cause moderately severe IPD. Here, we report a moderate CFR (12–16%) for these serotypes, consistent with trends reported elsewhere. This finding adds to the limited data on these serotypes (7, 19, 20). Other serotypes known to have moderate severity CFRs similarly found in our study included serotypes 4, 5, 7F, 8, and 12F. The clinical outcome of some serotypes like 11A, 15B, and 22F in IPD is most often unfavorable (21). Congruously, serotypes 31, 6A, and 11A were the three most lethal serotypes in the study. Being the serotype with the highest CFR (31.4%), serotype 31 had a significantly higher risk of death due to IPD (Pooled RR = 1.75 [95%CI: 1.06–2.89]). IPD cases with the second and third-highest CFR serotypes (serotype 11A and serotype 6A) were also significantly more likely to die. Serotype 15B, despite showing a moderate-to-severe CFR with an elevated risk of death was not associated with a statistically significant pooled relative risk (Table 3).

Serotypes 19F and 19A were the most represented serotypes in the study. These serotypes have been reported as the two most frequent serotypes across pediatric and adult populations (22). Elsewhere, serotype 19A was identified as the most common in the pneumococcal carriage and the second most common invasive serotype among the conjugate vaccine serotypes (23). Serotype 19A has been associated with antibiotic resistance. Specifically, penicillin non-susceptibility and multidrug resistance were observed more significantly in serotype 19A isolates than in non-19A isolates in IPD (24). Similarly, high rates of multidrug resistance and good nasopharyngeal colonization efficiency of serotype 19F isolates have been demonstrated in other studies (25, 26). Serotype 19F has been linked to severe disease outcomes and high case fatality rates (27). Observing Serotype 19F as one of the serotypes with a significantly higher relative risk of death is worrisome owing to the aforementioned dynamics in carriage, invasiveness, antimicrobial resistance, and mortality. Serotype 17F was the least frequent in our study. The rarity of serotype 17F is widely noted in studies and global surveillance data on pneumococcal serotypes (28–30).

The findings of this systematic review showed a wide geographical distribution of studies focusing on pneumococcal serotypes and IPD outcomes, specifically mortality. Our results highlight a strong European representation in pneumococcal serotype studies, aligning with extensive surveillance systems in Europe (31). This was followed by Asia, North America, and Africa, reflecting global disparities in research focus. Unsurprisingly, Europe currently has the highest vaccine coverage (86% in 2024) according to the WHO data report. The African region, Eastern Mediterranean region, and Western Pacific region have relatively lower vaccine coverage (70, 52, and 26%, respectively). Pneumococcal serotype and IPD surveillance heavily rely on comprehensive network of clinical, epidemiological and laboratory investigations, which are costly to establish and maintain. Hence the underrepresentation of some geographical regions, particularly Africa, may be explained by economic challenges facing LMICs in the region and other regions. Although current vaccine policies have ensured substantial reduction of IPD in some African LMICs such as South Africa (32), Kenya (33), and Gambia (34), limited data on serotype-specific monitoring in IPD could stall the benefits of current vaccine developments considering the potential vaccine-driven shift in serotype distribution. Notably, there was a consistent publication trend, where a remarkable increase in publications after 2015 was observed (Table 1). This is likely due to advancements in diagnostic technologies and global efforts to evaluate available PCVs and PPSV23.

The study periods suggest robust longitudinal data collection, with studies spanning decades enhancing the validity of temporal trends. However, the underrepresentation of Africa (a region with a disproportionate burden of pneumococcal disease morbidity and mortality) and South America in published data precipitates potential gaps in pneumococcal disease surveillance and research in low-resource settings. Future research should address these geographical imbalances to provide a more comprehensive global understanding of serotype influence on IPD outcomes. Such efforts could improve pneumococcal vaccine policy effectiveness across diverse regions.

We observed a significantly higher risk of death for VT compared to NVT [Q_*M*_ (1) = 7.12, *p* = 0.0076]. A sensitivity analysis to assess the subgroup difference-robustness indicated significant reliability in the difference identified. Thus, excluding the NVT subgroup, the meta-analysis for VT alone revealed a pooled log risk ratio of 0.0173 (95% CI: -0.0688 to 0.1034; *p* = 0.6942) with moderate heterogeneity (τ^2^ = 0.1340; I^2^ = 48.48%). While vaccine serotypes were associated with a higher overall risk of death, the effect size varied when analyzed independently, highlighting the complexity of serotype-specific mortality risks. These results have important clinical and epidemiological implications. Vaccine serotypes are often associated with higher invasiveness and pathogenicity, which could explain the increased mortality risk observed in this study and others (7, 18, 20). Despite the success of PCVs in reducing the incidence of IPD caused by vaccine serotypes (35), our study highlights the continued burden of VT-associated mortality. This underscores the need for ongoing surveillance and evaluation of vaccine efficacy. In other words, we should aim for targeted public health interventions to mitigate the burden of vaccine serotypes, even as efforts continue to address non-vaccine serotypes.

Additionally, the lack of significant relative risk of death among several VT may suggest that current PCVs and PPSV23 effectively reduce the common serotypes. However, the potential for serotype replacement phenomena, where non-vaccine serotypes become more prevalent, necessitates vaccine design and implementation vigilance. More so, NVT as shown in this study (serotype 6C, 15A, and serotype 31) pose potential threats as far as IPD outcome is concerned. Reasons for the higher risk of death associated with serotype 31 is not fully understood, however, some factors could be considered in explaining this observation. These factors may include serotype 31’s intrinsic virulence factors such as harboring antibiotic resistance genes and biofilm-forming abilities, which can exacerbate IPD outcome and potentially contribute to the higher case fatality rates (as usually seen with other serotypes like 11A). Serotype 31 has been identified as a relatively good biofilm former (36), implicating an enhanced evasion potential against host’s complement-mediated immunity by this serotype (37).

Expanding vaccine coverage to include additional serotypes could further reduce the overall burden of pneumococcal disease. Our crucial finding about risk of death for some NVTs is vital to inform their inclusion recent higher-valent formulations. Acknowledging that epidemiological data is key in selecting vaccine serotypes, our results, as well as the individual studies included provide enough evidence to consider incorporating serotype 31, and 15A into vaccine formulations.

The variability in study design, population demographics, and clinical settings across included studies limits our analysis. Additionally, we could not account for all potential confounding factors (e.g., comorbidities, age, and gender). Future research should focus on longitudinal studies to assess the long-term impact of vaccination programs on both VT and NVT.

**FIGURE 10:**
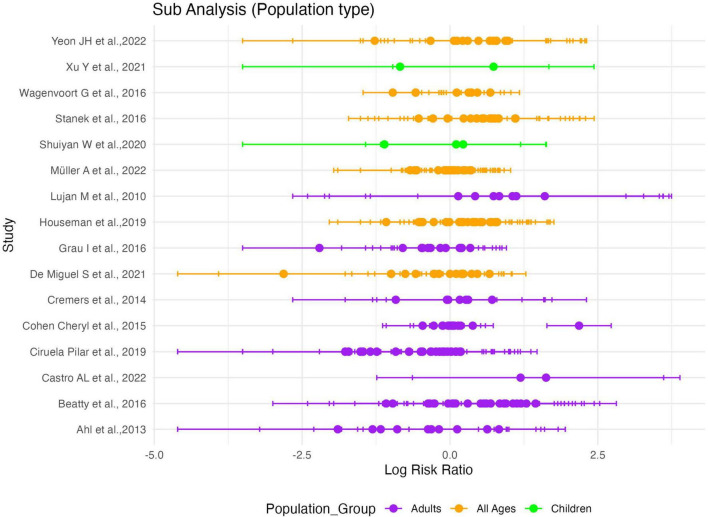
Sub-group meta-analysis showing no difference in serotype-specific relative risk estimates among population groups serotypes (NVT). Plot of serotype-specific risk ratios across all sixteen (16) studies included in the meta-analysis, stratified by population age groups. Purple dots on the plot represent the adult subgroup. Orange dots represent all age subgroups, green dots represent children subgroup.

Focussing on variations across three population groups (children, adults, and all ages) on *S. pneumoniae* serotype association to the risk of death, subgroup meta-regression did not reveal significant differences in risk estimates between population groups (Figure 10). This finding suggests a consistent lack of effect across demographic contexts. The observed heterogeneity in the “adults” and “all ages” subgroups (I^2^ = 49.61 and 37.34%, respectively) highlights the variability in study characteristics and/or populations not fully captured by the analysis. In contrast, the “children” subgroup demonstrated no heterogeneity, likely due to the smaller number of studies and more homogeneous study populations. It is important to note that only a few studies that report IPD in children met the inclusion criteria for meta-analysis; most of the studies did not report serotype 14 (reference serotype) in children and hence could not be used in computing relative risks.

It is also worth mentioning that the pooled estimates of some serotypes in the study are based on small numbers and, hence, subject to some degree of uncertainty. For example, IPD-related deaths were less frequent in some Asian studies that focused on children (38, 39).

As a limitation, this study did not assess the association between serotype-specific risk of death, complications, and the presence of comorbidities in IPD. However, it is well-known that IPD outcomes can be affected by both serotype and host characteristics (40, 41). The mortality associated with invasive pneumococcal disease can be largely affected by complications and comorbidities in IPD. For instance, CFRs in patients with pneumococcal pneumonia who show signs of septic shock are about 20% more than the CFRs of those without septic shock (42). For this reason, more studies investigating the association of serotypes with disease severity and outcomes are imperative to better understand how serotype influences aspects of pneumococcal epidemiology. Additionally, understanding regional variations in pneumococcal serotypes and their patterns greatly informs the development of more effective and targeted treatment strategies. Hence, there is the need to strengthen capacity for more region-specific studies in LMICs of Africa, and South America where data on serotype-specific case fatalities in IPD is scanty.

## 5 Conclusion

The evidence from our study confirms the stable role of pneumococcal serotype in determining the clinical outcomes of invasive pneumococcal disease. Our understanding of the patterns of serotype-specific risk of death in IPD will be beneficial in surveillance and public health preventive interventions. Specifically, potential benefits lie within our ability to explore the use of pneumococcal vaccines that have increased coverage to target serotypes with a higher risk of death. Our findings provide an informed roadmap for pneumococcal vaccine development, and re-evaluating clinical management strategies to ensure better prediction of IPD outcomes by health care providers. Despite VT serotypes generally having relatively higher death risks, It is necessary to clinically manage IPD patients with high mortality risk-NVT serotypes such as serotype 31, more critically. Given that some serotypes not included in currently available PCVs and PPSV23 pose a higher risk of death due to invasive pneumococcal disease (IPD), it is crucial to consider expanding the pneumococcal vaccine to include these high-risk serotypes and maximize vaccine impact. Additionally, public health strategies should prioritize the development and introduction of vaccines targeting additional serotypes to reduce mortality from IPD. Strengthening awareness and prevention efforts, especially in high-risk populations, will further mitigate the impact of these invasive infections. Finally, there is an alarming rate of global disparity in IPD and serotype research focus. Hence, efforts to build research capacity via funded training programs, longitudinal studies, and trials incorporating new vaccine candidates, especially in low-resource regions such as Africa, South America and the Western Pacific, are imperative to bridge the gap.

## Data Availability

The original contributions presented in the study are included in the article/Supplementary material, further inquiries can be directed to the corresponding author.
