# Supplementary material for: Pneumococcal serotypes and their association with death risk in invasive pneumococcal disease: a systematic review and meta-analysis
Source: Front Med (Lausanne). 2025 May 14;12:1566502. doi: 10.3389/fmed.2025.1566502 (PMC12116315; doi:10.3389/fmed.2025.1566502)
Supplement: Supplementary file 1 [file Table_1.docx]

**Supplementary Tables and Figures**

| **Table S1. Studies used for meta-analysis and their respective serotype-specific cases and fatalities** | | | |
| --- | --- | --- | --- |
| Serotype | Deaths | Cases | Study |
| S1 | 0 | 14 | Ahl et al.,2013 |
|  | 1 | 28 | Beatty et al., 2016 |
|  | 0 | 13 | Ciruela Pilar et al., 2019 |
|  | 246 | 651 | Cohen Cheryl et al., 2015 |
|  | 4 | 417 | De Miguel S et al., 2021 |
|  | 4 | 26 | Gaensbauer JT et al., 2016 |
|  | 3 | 115 | Grau I et al., 2016 |
|  | 12 | 198 | Houseman et al.,2019 |
|  | 16 | 111 | Hu T et al., 2021a |
|  | 8 | 326 | Hu T et al., 2021b |
|  | 91 | 282 | Müller A et al., 2022(data from suppl) |
|  | 4 | 58 | Skoczyńska A et al., 2014 |
|  | 5 | 52 | Stanek et al., 2016 |
|  | 6 | 153 | Verhaegen J et al., 2014 |
|  | 143 | 735 | Von Mollendorf C et al., 2023 |
|  | 23 | 384 | Wagenvoort G et al., 2016 |
| S3 | 11 | 36 | Ahl et al.,2013 |
|  | 434 | 1625 | Amin-Chowdhury Z et al., 2020 |
|  | 7 | 20 | Aydin MA et al., 2023 |
|  | 32 | 116 | Beatty et al., 2016 |
|  | 63 | 270 | Calvo-Silveria et al., 2024 |
|  | 6 | 13 | Castro AL et al., 2022 |
|  | 32 | 126 | Ciruela Pilar et al., 2019 |
|  | 98 | 284 | Cohen Cheryl et al., 2015 |
|  | 6 | 30 | Cremers et al., 2014 |
|  | 113 | 605 | De Miguel S et al., 2021 |
|  | 51 | 183 | Grau I et al., 2016 |
|  | 56 | 189 | Houseman et al.,2019 |
|  | 12 | 111 | Hu T et al., 2021a |
|  | 7 | 326 | Hu T et al., 2021b |
|  | 8 | 37 | Lujan M et al., 2010 |
|  | 98 | 290 | Müller A et al., 2022(data from suppl) |
|  | 23 | 89 | Stanek et al., 2016 |
|  | 32 | 111 | Verhaegen J et al., 2014 |
|  | 64 | 292 | Wagenvoort G et al., 2016 |
|  | 34 | 153 | Yeon JH et al.,2022 |
| S4 | 3 | 55 | Ahl et al.,2013 |
|  | 17 | 164 | Beatty et al., 2016 |
|  | 0 | 10 | Ciruela Pilar et al., 2019 |
|  | 83 | 289 | Cohen Cheryl et al., 2015 |
|  | 5 | 26 | Cremers et al., 2014 |
|  | 12 | 103 | De Miguel S et al., 2021 |
|  | 13 | 67 | Grau I et al., 2016 |
|  | 7 | 42 | Houseman et al.,2019 |
|  | 2 | 15 | Lujan M et al., 2010 |
|  | 68 | 223 | Müller A et al., 2022(data from suppl) |
|  | 26 | 71 | Skoczyńska A et al., 2014 |
|  | 11 | 55 | Stanek et al., 2016 |
| S5 | 8 | 247 | Beatty et al., 2016 |
|  | 11 | 53 | Cohen Cheryl et al., 2015 |
|  | 1 | 116 | De Miguel S et al., 2021 |
|  | 4 | 17 | Gaensbauer JT et al., 2016 |
|  | 5 | 49 | Grau I et al., 2016 |
|  | 1 | 111 | Hu T et al., 2021a |
|  | 1 | 326 | Hu T et al., 2021b |
|  | 2 | 16 | Lujan M et al., 2010 |
|  | 8 | 28 | Müller A et al., 2022(data from suppl) |
|  | 17 | 149 | Zurawska JH et al.,2013 |
| S6A | 3 | 20 | Ahl et al.,2013 |
|  | 12 | 35 | Beatty et al., 2016 |
|  | 89 | 269 | Cohen Cheryl et al., 2015 |
|  | 7 | 33 | Grau I et al., 2016 |
|  | 14 | 40 | Houseman et al.,2019 |
|  | 3 | 111 | Hu T et al., 2021a |
|  | 15 | 326 | Hu T et al., 2021b |
|  | 54 | 121 | Müller A et al., 2022(data from suppl) |
|  | 3 | 12 | Stanek et al., 2016 |
| S6B | 1 | 24 | Ahl et al.,2013 |
|  | 7 | 32 | Beatty et al., 2016 |
|  | 2 | 10 | Ciruela Pilar et al., 2019 |
|  | 64 | 202 | Cohen Cheryl et al., 2015 |
|  | 6 | 42 | Grau I et al., 2016 |
|  | 6 | 24 | Shuiyan W et al.,2020 |
|  | 12 | 31 | Stanek et al., 2016 |
|  | 6 | 11 | Verhaegen J et al., 2014 |
|  | 27 | 118 | Wagenvoort G et al., 2016 |
|  | 1 | 13 | Yeon JH et al.,2022 |
| S6C | 12 | 44 | Ciruela Pilar et al., 2019 |
|  | 29 | 155 | De Miguel S et al., 2021 |
|  | 16 | 58 | Houseman et al.,2019 |
|  | 13 | 44 | Müller A et al., 2022(data from suppl) |
|  | 7 | 24 | Stanek et al., 2016 |
|  | 7 | 30 | Yeon JH et al.,2022 |
| S7F | 6 | 65 | Ahl et al.,2013 |
|  | 7 | 77 | Beatty et al., 2016 |
|  | 5 | 24 | Ciruela Pilar et al., 2019 |
|  | 15 | 73 | Cohen Cheryl et al., 2015 |
|  | 2 | 34 | Cremers et al., 2014 |
|  | 16 | 286 | De Miguel S et al., 2021 |
|  | 14 | 99 | Grau I et al., 2016 |
|  | 30 | 222 | Houseman et al.,2019 |
|  | 10 | 111 | Hu T et al., 2021a |
|  | 20 | 326 | Hu T et al., 2021b |
|  | 1 | 20 | Lujan M et al., 2010 |
|  | 23 | 81 | Müller A et al., 2022(data from suppl) |
|  | 6 | 79 | Stanek et al., 2016 |
|  | 20 | 159 | Verhaegen J et al., 2014 |
|  | 54 | 600 | Wagenvoort G et al., 2016 |
| S8 | 2 | 18 | Ahl et al.,2013 |
|  | 291 | 3365 | Amin-Chowdhury Z et al., 2020 |
|  | 11 | 152 | Beatty et al., 2016 |
|  | 6 | 91 | Ciruela Pilar et al., 2019 |
|  | 52 | 209 | Cohen Cheryl et al., 2015 |
|  | 6 | 43 | Cremers et al., 2014 |
|  | 71 | 990 | De Miguel S et al., 2021 |
|  | 25 | 231 | Houseman et al.,2019 |
|  | 1 | 11 | Lujan M et al., 2010 |
|  | 156 | 507 | Müller A et al., 2022(data from suppl) |
|  | 5 | 31 | Stanek et al., 2016 |
|  | 39 | 433 | Wagenvoort G et al., 2016 |
| S9N | 212 | 1128 | Amin-Chowdhury Z et al., 2020 |
|  | 4 | 40 | Beatty et al., 2016 |
|  | 5 | 36 | Ciruela Pilar et al., 2019 |
|  | 26 | 89 | Cohen Cheryl et al., 2015 |
|  | 29 | 172 | De Miguel S et al., 2021 |
|  | 15 | 57 | Houseman et al.,2019 |
|  | 37 | 127 | Müller A et al., 2022(data from suppl) |
|  | 5 | 19 | Stanek et al., 2016 |
| S9V | 4 | 41 | Ahl et al.,2013 |
|  | 4 | 60 | Beatty et al., 2016 |
|  | 4 | 21 | Ciruela Pilar et al., 2019 |
|  | 45 | 140 | Cohen Cheryl et al., 2015 |
|  | 4 | 23 | Cremers et al., 2014 |
|  | 15 | 82 | De Miguel S et al., 2021 |
|  | 24 | 89 | Grau I et al., 2016 |
|  | 10 | 37 | Houseman et al.,2019 |
|  | 1 | 10 | Lujan M et al., 2010 |
|  | 7 | 41 | Müller A et al., 2022(data from suppl) |
|  | 7 | 57 | Stanek et al., 2016 |
| S10A | 4 | 28 | Ciruela Pilar et al., 2019 |
|  | 10 | 118 | De Miguel S et al., 2021 |
|  | 6 | 34 | Houseman et al.,2019 |
|  | 48 | 121 | Müller A et al., 2022(data from suppl) |
| S11A | 118 | 400 | Amin-Chowdhury Z et al., 2020 |
|  | 14 | 48 | Beatty et al., 2016 |
|  | 7 | 32 | Ciruela Pilar et al., 2019 |
|  | 43 | 179 | De Miguel S et al., 2021 |
|  | 18 | 47 | Houseman et al.,2019 |
|  | 21 | 48 | Müller A et al., 2022(data from suppl) |
|  | 13 | 25 | Skoczyńska A et al., 2014 |
|  | 19 | 75 | Wagenvoort G et al., 2016 |
|  | 14 | 49 | Yeon JH et al.,2022 |
| S12F | 209 | 2086 | Amin-Chowdhury Z et al., 2020 |
|  | 5 | 51 | Beatty et al., 2016 |
|  | 3 | 73 | Ciruela Pilar et al., 2019 |
|  | 60 | 175 | Cohen Cheryl et al., 2015 |
|  | 11 | 195 | De Miguel S et al., 2021 |
|  | 10 | 96 | Houseman et al.,2019 |
|  | 106 | 375 | Müller A et al., 2022(data from suppl) |
|  | 17 | 120 | Shimbashi R et al., 2019 |
|  | 6 | 96 | Verhaegen J et al., 2014 |
|  | 1 | 33 | Yeon JH et al.,2022 |
| S14 | 8 | 60 | Ahl et al.,2013 |
|  | 8 | 85 | Beatty et al., 2016 |
|  | 1 | 11 | Castro AL et al., 2022 |
|  | 18 | 79 | Ciruela Pilar et al., 2019 |
|  | 83 | 253 | Cohen Cheryl et al., 2015 |
|  | 5 | 34 | Cremers et al., 2014 |
|  | 20 | 132 | De Miguel S et al., 2021 |
|  | 28 | 123 | Grau I et al., 2016 |
|  | 8 | 45 | Houseman et al.,2019 |
|  | 1 | 23 | Lujan M et al., 2010 |
|  | 24 | 77 | Müller A et al., 2022(data from suppl) |
|  | 4 | 20 | Shuiyan W et al.,2020 |
|  | 6 | 47 | Stanek et al., 2016 |
|  | 64 | 401 | Wagenvoort G et al., 2016 |
|  | 2 | 12 | Xu Y et al., 2021 |
|  | 3 | 28 | Yeon JH et al.,2022 |
| S15A | 171 | 758 | Amin-Chowdhury Z et al., 2020 |
|  | 3 | 11 | Beatty et al., 2016 |
|  | 8 | 32 | Ciruela Pilar et al., 2019 |
|  | 25 | 131 | De Miguel S et al., 2021 |
|  | 11 | 46 | Houseman et al.,2019 |
|  | 60 | 154 | Müller A et al., 2022(data from suppl) |
|  | 8 | 34 | Yeon JH et al.,2022 |
| S15B | 4 | 10 | Beatty et al., 2016 |
|  | 11 | 95 | De Miguel S et al., 2021 |
|  | 48 | 147 | Müller A et al., 2022(data from suppl) |
|  | 4 | 23 | Yeon JH et al.,2022 |
| S16F | 3 | 19 | Beatty et al., 2016 |
|  | 7 | 30 | Ciruela Pilar et al., 2019 |
|  | 8 | 26 | Houseman et al.,2019 |
|  | 64 | 178 | Müller A et al., 2022(data from suppl) |
| S17F | 5 | 16 | Beatty et al., 2016 |
|  | 1 | 17 | Ciruela Pilar et al., 2019 |
|  | 29 | 113 | Müller A et al., 2022(data from suppl) |
| S18C | 4 | 16 | Ahl et al.,2013 |
|  | 5 | 30 | Beatty et al., 2016 |
|  | 1 | 11 | Ciruela Pilar et al., 2019 |
|  | 24 | 68 | Cohen Cheryl et al., 2015 |
|  | 6 | 38 | Grau I et al., 2016 |
|  | 3 | 27 | Houseman et al.,2019 |
|  | 17 | 48 | Müller A et al., 2022(data from suppl) |
|  | 2 | 26 | Skoczyńska A et al., 2014 |
|  | 3 | 11 | Stanek et al., 2016 |
|  | 20 | 87 | Wagenvoort G et al., 2016 |
| S19A | 170 | 946 | Amin-Chowdhury Z et al., 2020 |
|  | 9 | 71 | Beatty et al., 2016 |
|  | 3 | 10 | Castro AL et al., 2022 |
|  | 1 | 27 | Chen-Yin er al., 2012 |
|  | 9 | 55 | Ciruela Pilar et al., 2019 |
|  | 151 | 443 | Cohen Cheryl et al., 2015 |
|  | 57 | 455 | De Miguel S et al., 2021 |
|  | 19 | 116 | Grau I et al., 2016 |
|  | 39 | 176 | Houseman et al.,2019 |
|  | 13 | 111 | Hu T et al., 2021a |
|  | 34 | 326 | Hu T et al., 2021b |
|  | 1 | 15 | Lujan M et al., 2010 |
|  | 3 | 20 | Makwana A et al.,2019 |
|  | 109 | 379 | Müller A et al., 2022(data from suppl) |
|  | 1 | 15 | Shuiyan W et al.,2020 |
|  | 9 | 41 | Stanek et al., 2016 |
|  | 22 | 143 | Verhaegen J et al., 2014 |
|  | 1 | 14 | Xu Y et al., 2021 |
|  | 10 | 69 | Yeon JH et al.,2022 |
| S19F | 4 | 16 | Ahl et al.,2013 |
|  | 6 | 32 | Beatty et al., 2016 |
|  | 3 | 18 | Ciruela Pilar et al., 2019 |
|  | 100 | 207 | Cohen Cheryl et al., 2015 |
|  | 3 | 10 | Cremers et al., 2014 |
|  | 19 | 87 | De Miguel S et al., 2021 |
|  | 24 | 75 | Grau I et al., 2016 |
|  | 14 | 39 | Houseman et al.,2019 |
|  | 70 | 178 | Müller A et al., 2022(data from suppl) |
|  | 4 | 18 | Shuiyan W et al.,2020 |
|  | 5 | 22 | Stanek et al., 2016 |
|  | 29 | 92 | Wagenvoort G et al., 2016 |
|  | 8 | 23 | Xu Y et al., 2021 |
|  | 4 | 19 | Yeon JH et al.,2022 |
| S20 | 1 | 14 | Beatty et al., 2016 |
|  | 8 | 36 | Houseman et al.,2019 |
|  | 3 | 19 | Müller A et al., 2022(data from suppl) |
|  | 42 | 189 | Stanek et al., 2016 |
|  | 4 | 35 | Yeon JH et al.,2022 |
| S22F | 193 | 1218 | Amin-Chowdhury Z et al., 2020 |
|  | 236 | 1528 | Amin-Chowdhury Z et al., 2021 |
|  | 16 | 92 | Beatty et al., 2016 |
|  | 6 | 53 | Ciruela Pilar et al., 2019 |
|  | 64 | 22 | Cohen Cheryl et al., 2015 |
|  | 36 | 238 | De Miguel S et al., 2021 |
|  | 32 | 154 | Houseman et al.,2019 |
|  | 2 | 13 | Marrie Thomas et al., 2018 |
|  | 45 | 111 | Müller A et al., 2022(data from suppl) |
|  | 9 | 24 | Skoczyńska A et al., 2014 |
|  | 11 | 61 | Verhaegen J et al., 2014 |
|  | 4 | 33 | Yeon JH et al.,2022 |
| S23A | 89 | 388 | Amin-Chowdhury Z et al., 2020 |
|  | 3 | 16 | Beatty et al., 2016 |
|  | 7 | 30 | Ciruela Pilar et al., 2019 |
|  | 16 | 87 | De Miguel S et al., 2021 |
|  | 12 | 46 | Houseman et al.,2019 |
|  | 23 | 80 | Müller A et al., 2022(data from suppl) |
|  | 2 | 10 | Stanek et al., 2016 |
|  | 8 | 60 | Yeon JH et al.,2022 |
| S23F | 0 | 25 | Ahl et al.,2013 |
|  | 8 | 33 | Beatty et al., 2016 |
|  | 99 | 252 | Cohen Cheryl et al., 2015 |
|  | 2 | 14 | Cremers et al., 2014 |
|  | 16 | 58 | Grau I et al., 2016 |
|  | 9 | 42 | Houseman et al.,2019 |
|  | 43 | 136 | Müller A et al., 2022(data from suppl) |
|  | 9 | 31 | Stanek et al., 2016 |
|  | 31 | 174 | Wagenvoort G et al., 2016 |
| S31 | 7 | 39 | Ciruela Pilar et al., 2019 |
|  | 31 | 105 | De Miguel S et al., 2021 |
|  | 15 | 38 | Houseman et al.,2019 |
|  | 5 | 13 | Stanek et al., 2016 |
| S33F | 109 | 632 | Amin-Chowdhury Z et al., 2020 |
|  | 128 | 776 | Amin-Chowdhury Z et al., 2021 |
|  | 2 | 31 | Beatty et al., 2016 |
|  | 1 | 19 | Ciruela Pilar et al., 2019 |
|  | 12 | 55 | Houseman et al.,2019 |
|  | 3 | 17 | Müller A et al., 2022(data from suppl) |
| S35B | 4 | 17 | Beatty et al., 2016 |
|  | 7 | 26 | Ciruela Pilar et al., 2019 |
|  | 18 | 96 | De Miguel S et al., 2021 |
|  | 33 | 105 | Müller A et al., 2022(data from suppl) |
|  | 2 | 11 | Stanek et al., 2016 |
|  | 12 | 44 | Yeon JH et al.,2022 |
| Table S1: A list of all sixteen (16) IPD studies selected for meta-analysis. The Table shows the cases and deaths of all 29 serotype per study. Studies in blue font are studies which reported on children/pediatric IPD. | | | |


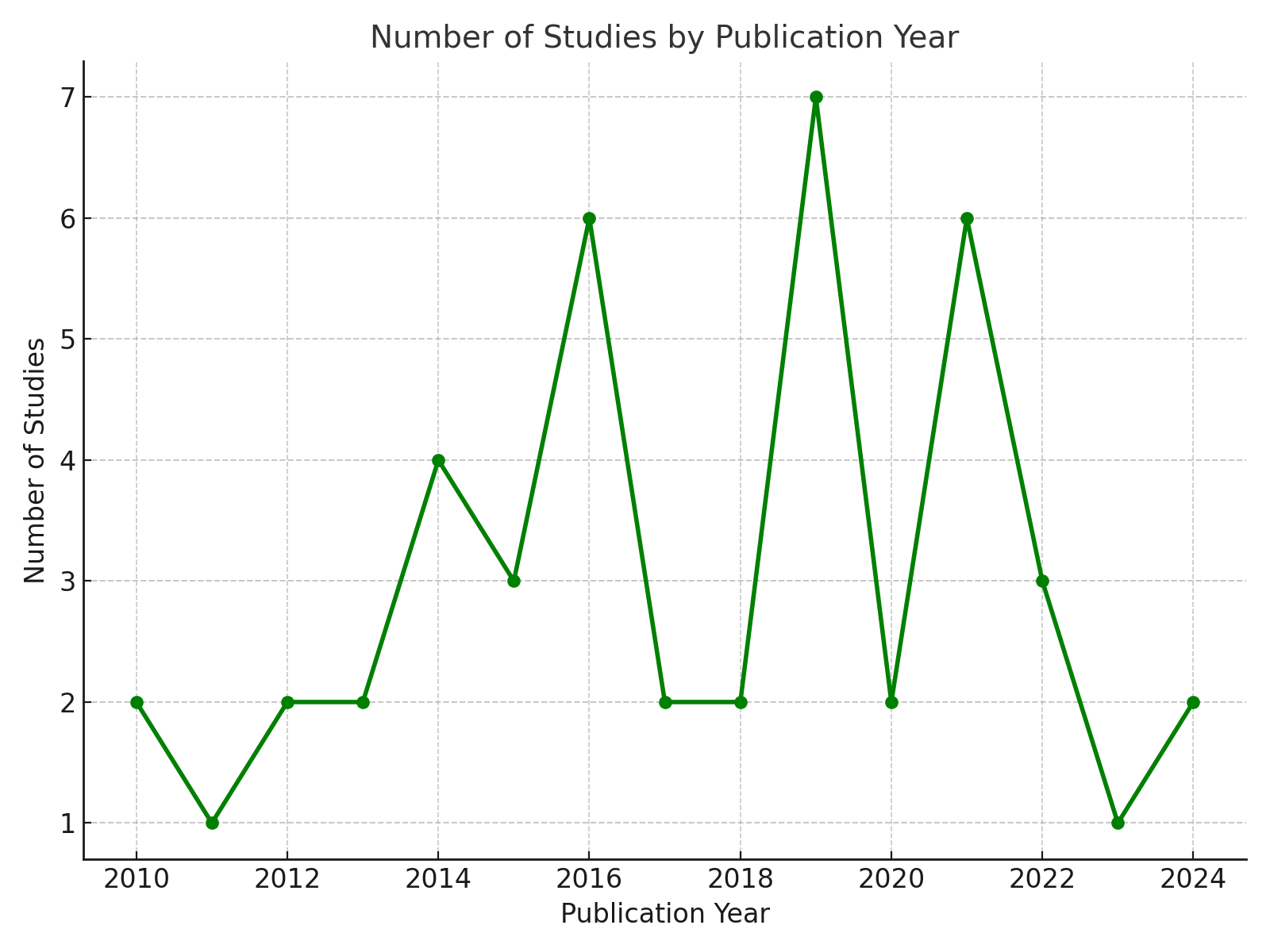


Supplementary Figure 1: The trend of publications focussing on serotype-specific case fatalities in IPD over the last decade
